# The impact of ivacaftor on sinonasal pathology in S1251N-mediated cystic fibrosis patients

**DOI:** 10.1371/journal.pone.0235638

**Published:** 2020-07-20

**Authors:** Romee Gostelie, Inge Stegeman, Gitte Berkers, Joost Bittermann, Ivonne Ligtenberg-van der Drift, Peter-Jan van Kipshagen, Karin de Winter - de Groot, Lucienne Speleman

**Affiliations:** 1 University Medical Center, Utrecht University, Utrecht, The Netherlands; 2 Department of Otorhinolaryngology, Head and Neck Surgery, University Medical Center, Utrecht University, Utrecht, The Netherlands; 3 Department of Pediatric Respiratory Medicine and Allergology, Cystic Fibrosis Center, University Medical Center, Utrecht University, Utrecht, The Netherlands; 4 Department of Pediatric Otorhinolaryngology, University Medical Center, Utrecht University, Utrecht, The Netherlands; 5 Department of Otorhinolaryngology, Beatrix Hospital, Gorinchem, The Netherlands; University of Catania, ITALY

## Abstract

**Importance:**

Sinonasal symptoms in patients suffering from cystic fibrosis can negatively influence the quality of life and sinuses can be a niche for pathogens causing infection and inflammation leading to a decrease of lung function. Ivacaftor, a potentiator of the Cystic Fibrosis Transmembrane Conductance Regulator protein, has shown improvement in pulmonary function in cystic fibrosis patients with different forms of class III gating mutations. However, the effects of ivacaftor on sinonasal pathology have hardly been studied.

**Objective:**

To determine the impact of ivacaftor therapy on sinonasal pathology in patients with cystic fibrosis with an S1251N mutation.

**Design:**

Prospective observational mono-center cohort study, between June 2015 and December 2016.

**Setting:**

A tertiary referral center in Utrecht, The Netherlands.

**Participants:**

Eight patients with cystic fibrosis with an S1251N mutation, treated with the potentiator ivacaftor were investigated.

**Exposures:**

Ivacaftor (Kalydeco, VX-770) therapy. Computed tomography imaging of paranasal sinuses. Nasal nitric oxide concentration measurements and nasal endoscopy.

**Main outcomes and measures:**

Primary outcome is opacification of paranasal sinuses examined with computed tomography scan analysis and scaled by the modified Lund-Mackay score before and one year after treatment. Secondary outcomes are nasal nitric oxide concentration levels, sinonasal symptoms and nasal endoscopic findings before and approximately two months and in some cases one year after treatment.

**Results:**

Computed tomography scan analysis showed a significant decrease in opacification of the majority of paranasal sinuses comparing the opacification score per paranasal sinus before and after one year of treatment with ivacaftor. Median nasal nitric oxide levels significantly improved from 220.00 (IQR:136.00–341.18) to 462.84 (IQR:233.17–636.25) (p = 0.017) parts per billion. Likewise, the majority of sinonasal symptoms and nasal endoscopic pathology decreased or resolved at two months after the use of ivacaftor.

**Conclusion and relevance:**

Ivacaftor appears to improve sinonasal outcome parameters and thereby sinonasal health in patients with cystic fibrosis with an S1251N mutation.

## Introduction

Cystic fibrosis (CF) is one of the most common autosomal recessive genetic disorders in the Caucasian population, with an incidence of approximately 1 in 4000–5500 live births in the Netherlands.[1,2] This life-shortening disease is caused by mutation in the *Cystic Fibrosis Transmembrane Conductance Regulator (CFTR)* gene, which encodes the CFTR protein, expressed as a chloride ion channel at the apical membranes of secretory epithelia. Due to loss of chloride transport, CF patients produce thick, viscous mucus in different organs.[3–5] Consequently, sinonasal disease is one of the systemic manifestations and occurs in almost all CF patients, with symptoms like upper respiratory tract infections, anosmia, headaches and nasal obstruction with or without nasal polyps.[6–8] Acknowledgment and treatment of upper airway problems in CF patients is important because it can cause a decrease in the quality of life.[6,7,9] Besides, it can be a niche for pathogens that can lead to pulmonary infections, which eventually leads to a decline in lung function and disease progression of CF.[7,9,10]

CFTR mutations can be grouped in six different disease classes. Class I-III mutations are associated with almost no functional CFTR protein and thereby a severe phenotype, with significantly higher incidence of hypoplasia or aplasia of the sinuses and more opacification in sinonasal area.[7,11] Class IV-VI mutations are associated with a reduced amount of functional CFTR and generally have a milder CF phenotype.[7]

To date, there is lack of conclusive evidence concerning the management of chronic sinonasal pathology in patients with CF. While there are no standardized guidelines, current treatment of sinonasal involvement in CF patients begins with conservative, medical management, including nasal saline irrigation, nasal steroids, systemic steroids, and topical or systemic antibiotics. When conservative intervention fails and sinonasal symptoms persist, functional endoscopic sinus surgery (FESS) is indicated.[4–6,9]

Most treatments for CF target the secondary effects of dysfunction of the CFTR protein. Since the discovery of the CFTR gene in 1989, innovative therapies which target the underlying basic defect in CFTR function have been developed.[3,12–14] One of these therapies, ivacaftor (VX-770 or Kalydeco), is a CFTR protein potentiator that can increase the opening time of the CFTR channel and therefore facilitate an increase in chloride transport in patients with a class III gating mutation or an Arg117H class IV mutation.[1,13,15–18]

Studies assessing the use of ivacaftor show significant improvement in pulmonary function.[19–22] Patients whom possessed at least one G551D CFTR mutation portray a decrease in pulmonary exacerbations, an improvement in the quality of life and body weight and a reduction of sweat chloride concentration, without severe adverse effects. Ivacaftor is approved for the treatment of CF in patients ages 6 months and older with gating mutations like the class III G551D mutation, the R117H mutation and some other splice mutations.[4,6, 23–25] Most studies have been done in G551D-mutated CF patients, although in vitro and vivo data suggest that ivacaftor may have potential benefit in other class III gating mutations.[16,17,26] Regarding sinus disease, few studies show significant improvement in sinonasal symptoms and resolution of opacification of paranasal sinuses after the treatment with ivacaftor.[27–29] To observe improvement in sinonasal symptoms, literature shows different measurements, of which computed tomography (CT) scan analysis, nasal nitric oxide (nNO) levels, sinonasal quality of life questionnaires and nasal endoscopy are the most important.[5,9,30–32]

Sinus CT is a widely utilized method of assessing the extent and staging of chronic rhinosinusitis. The most commonly used scoring system for the severity of rhinosinusitis is the Lund-Mackay (L-M) score, or the modified L-M score in patients with a different degree of sinus development.[5,31,33,34]

Nitric oxide (NO) is a free radical gas produced within the respiratory tract by the conversion of the amino acid L-arginine to L-citrulline via three forms of the enzyme NO synthase. NO occurs in high concentration in the upper respiratory tract and can be detected orally in exhaled air or measured directly from the nasal cavity and paranasal sinuses.[30,35] Previous studies show that nasal NO levels are significantly lower in patients with CF compared with healthy controls.[36–40]

To date, limited literature is available regarding the impact of ivacaftor in class III-mutated CF patients with sinonasal symptoms. Given the fact that ivacaftor potentiates defective chloride channels throughout various tissues, further research of ivacaftor in patients suffering from CF can possibly explore the decrease of sinonasal problems.[9,16]

Within this study we aim to evaluate the effect of ivacaftor in S125N-mediated CF patients on sinonasal health through studying CT scans of paranasal sinuses, nNO levels, sinonasal symptoms and nasal endoscopic findings.

## Methods

### Patients characteristics

Between June 2015 and December 2016, eight CF patients with an S1251N class III gating mutation were treated in the University Medical Centre Utrecht (UMCU), the Netherlands. Patients in whom treating physician felt ivacaftor would be appropriate as a part of their treatment plan, with ivacaftor 150mg two times a day, were included to this retrospective cohort study. The possession of an S1251N mutation was used as a criteria to determine which patients received ivacaftor. This decision was made as part of routine clinical care. The study protocol was approved by the Medical Ethical Committee of the Utrecht Medical Centre (METC Utrecht). Various clinical and sociodemographic data were collected from patient records, such as, age, gender, additional medication, and history of sinus surgery. Additionally, nasopharyngeal swab samples and sputum cultures or oropharyngeal swab samples were documented. The need for the consent of parents or guardians of the children included in this study was waived by the ethical committee.

### Computed tomography

All patients underwent a CT scan of the paranasal sinuses before and one year after ivacaftor therapy. The modified L-M score was used to analyze paranasal sinus opacification because of the high prevalence of non-developed paranasal sinuses in children with CF.^31^ This validated grading system scores every sinonasal part separately, with the following grade: 0: normal, 1: partial opacification and, 2: total opacification. Moreover, the ostiomeatal complex (OMC) was graded as 0: patent or 2: occluded. The maxillary sinus, anterior ethmoid, posterior ethmoid, sphenoid and frontal sinus of both sides were scored independently by four otorhinolaryngologists. They were blinded for patients characteristics and scored all the CT scans before and after the introduction of ivacaftor randomly. Using this method it was possible to compare the opacification (L-M) score per paranasal sinus before and after one year of treatment with ivacaftor. In general a lower L-M score often means a less severe sinonasal disease burden.[34,41]

If there was disagreement in the scoring of absence of paranasal sinuses, the sinus was not included in the analyses if two or more of the four otorhinolaryngologists indicated that the paranasal sinus was absent.

### Nasal nitric oxide measurement

Nasal NO levels were measured before and two months after the application of ivacaftor therapy, using a Chemiluminescence analyzer. A Teflon ‘nasal olive’ tube was inserted in one of the nostrils of the patient, to achieve an airtight seal, ensuring air could only leave the nostril via the central channel of the tube. Through the nasal tube, expirated air was collected with a constant flow rate, while patients exhaled slowly through the mouth against an expiratory resistance with a standardized positive pressure. Through this process, closure of the velum was achieved to measure only nNO concentrations in the upper airway region and not NO levels of the lower respiratory tract. nNO concentrations were recorded when the values reached a plateau of NO concentration. Measurements were done in triplicate, mean nNO values were calculated.

### Sinonasal symptoms and nasal endoscopic findings

Chronic rhinosinusitis related symptoms were recorded on a four point scale to indicate the severity of symptoms that affected the patient, with higher scores indicating higher severity. Information about the following symptoms: rhinorrhea, nasal obstruction, postnasal drip, facial pain, headache, and anosmia or hyposmia was retrospectively recorded based on the charts of the patients. For every sinonasal symptom, it was calculated how many of the included patients had this symptom before and after two months of ivacaftor use, as well as in which severity. The modified Lund-Kennedy score was used to score endoscopic findings.[42,43] Information about nasal polyps, nasal discharge, and edema was collected per patient before and after two months of ivacaftor therapy.

### Data and statistics

Statistical analysis of the data was performed using SPSS for Windows version 25. Descriptive statistics were used for the clinical and sociodemographic data of the participants. After assessing the distribution of the outcomes, comparison of the degree of paranasal sinus opacification with or without ivacaftor use was investigated with the non-parametric Wilcoxon Signed Rank test. L-M scores were done by four otorhinolaryngologists, therefore, we decided to do a sensitivity analysis in which we analyzed the lowest L-M score (best case) as well as the highest L-M score (worst case) given by the otorhinolaryngologists per sinonasal part, before and after one year of ivacaftor use. With the Kendall coefficient of concordance we calculated the interrater variability between the four otorhinolaryngologists. A higher value of Kendall’s coefficient means that the otolaryngologists applied essentially the same standard when scoring the opacification of the sinuses. Difference in nNO levels was tested with the Wilcoxon Signed Rank test. P-values of <0.05 were considered as statistically significant. Because of the low number of patients and the missing values in reported symptoms and endoscopic findings we did not perform statistical analysis for symptom or endoscopic reduction.

## Results

### Patients

Five out of eight patients were male (63%) and the median age at start of the application of ivacaftor was 16 years (range 9–26). Two patients had multiple sinus surgeries prior to ivacaftor treatment (25%) (Table 1). None of the patients had sinus surgery during the period of ivacaftor use. During ivacaftor treatment, two patients received systemic steroids. One used steroids for approximately one week, because of an exacerbation of CF, probably because of allergic bronchopulmonary aspergillosis (ABPA). The other used steroids for a longer period of time because of symptoms of arthritis. In three patients, Pseudomonas aeruginosa was cultured from the upper airways (nasopharyngeal swabs) before the use of ivacaftor. In two of them, no micro-organism was cultured from the upper airways after ivacaftor use. In one of these patients, a Staphylococcus aureus was cultured after the use of ivacaftor therapy. Staphylococcus aureus was also cultured from the upper airways in one patient before ivacaftor use. After the use of ivacaftor no micro-organism was cultured in this patient. Aspergillus fumigatus was cultured from the lower airways in five of the eight included patients before ivacaftor use. After the use of ivacaftor, in only one of these five patients Aspergillus fumigatus was cultured (S1 Table). One patient passed away a few months after starting with ivacaftor due to a Ecstasy (3,4-methyleendioxymethamfetamine) recreational drug overdose. Therefore, this patient could not receive a routine CT scan after one year and was excluded from analysis of the L-M scores.

**Table 1:** Patients characteristics.

Parameter	Total (No. = 8)
*Male*, *No*. *(%)*	5 (63)
*Age*, *years (median*, *IQR range)*	16 (9–26)
*Surgical history FESS*, *No*. *(%)*	2 (25)
*Genotype*	
ΔF508/S1251N, No. (%)	7 (88)
A455E/S1251N, No. (%)	1 (12)
*Other medication beside ivacaftor*	
Nasal steroids, No. (%)	6 (75)
Nasal saline irrigation, No. (%)	2 (25)
Systemic antibiotics, No. (%)	7 (88)
Systemic steroids, No. (%)	2 (25)

Abbreviations: No.: number of patients, FESS: functional endoscopic sinus surgery, IQR: interquartile range

### Computed tomography

**Fig 1:**
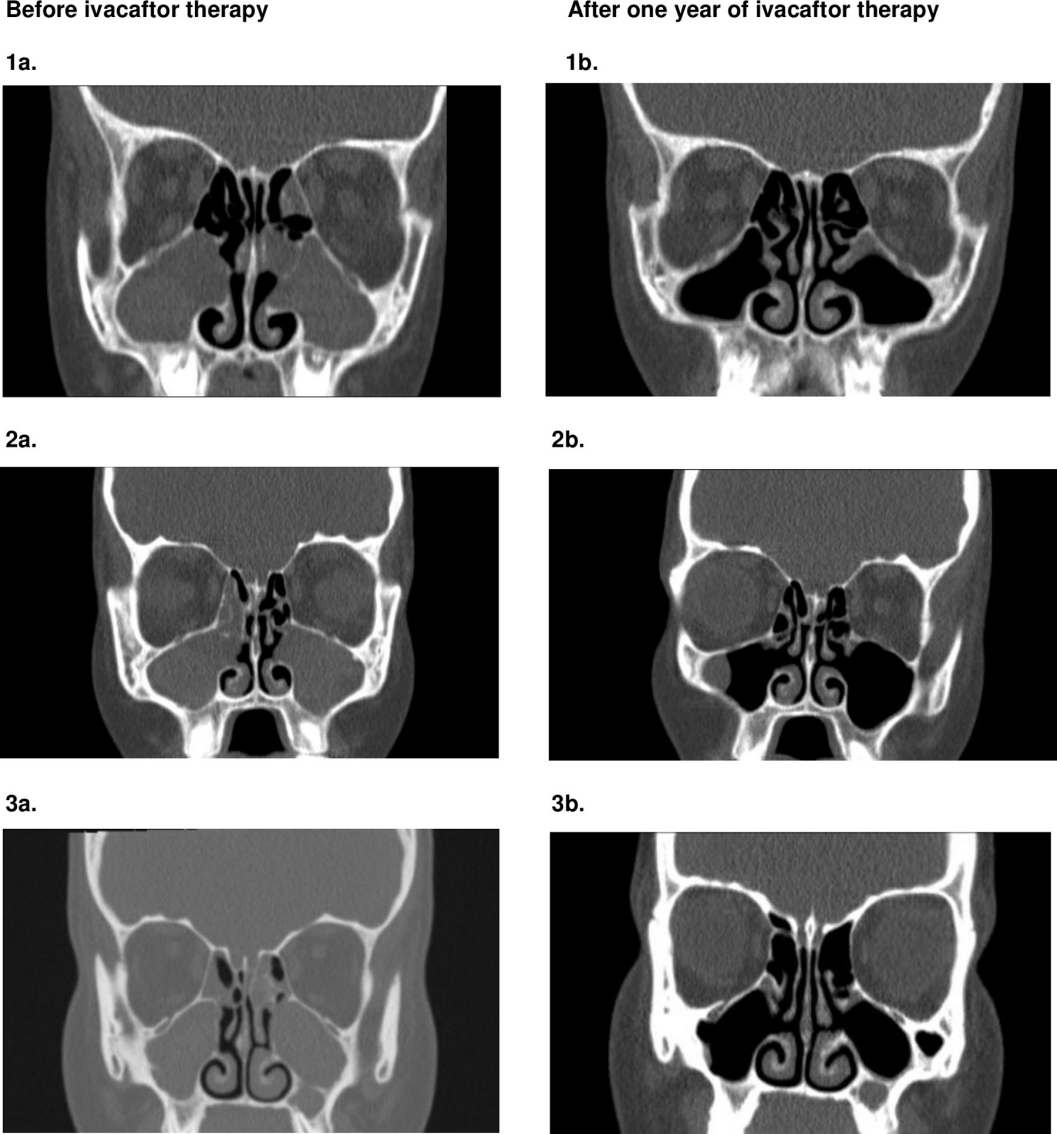
A couple of CT scans before and after one year of ivacaftor therapy.

For the maxillary sinus and anterior ethmoid there was a statistical significant decrease in sinus opacification on both sides after one year ivacaftor use, see Fig 1 and Table 2. Furthermore, the frontal sinus and OMC (Table 2) showed an improvement in sinus opacification and patency of the complex after one year of ivacaftor therapy for the right side of the frontal sinus and the left side of the OMC. The other parts of these sinonasal components showed no statistical significant improvement. For the posterior ethmoid and the sphenoid the differences between opacification before and after ivacaftor therapy were smaller. No statistical significant improvement in opacification was seen for these paranasal sinuses (Tables 2 and S2–S5). The interrater variability between the opacification scores of the four otolaryngologists before ivacaftor use varied between 0,061 and 0,505 and after ivacaftor use between 0,000 and 0,333 (Table 2).

**Table 2:** Opacification of the paranasal sinuses before and after one year of ivacaftor therapy.

Paranasal sinus component	Pre ivacaftor^∞^		Post ivacaftor^#^		p—value^×^	p—value^α^	KCC	
	*Best case*^*Δ*^ *(25*^*th*^*-75*^*th*^ *IQR)*	*Worst case*^*∑*^ *(25*^*th*^*-75*^*th*^ *IQR)*	*Best case*^*Δ*^ *(25*^*th*^*-75*^*th*^ *IQR)*	*Worst case*^*∑*^ *(25*^*th*^*-75*^*th*^ *IQR)*			Pre	Post
***Maxillary sinus right***	2.00 (1.00–2.00)	2.00 (2.00–2.00)	0.00 (0.00–0.00)	0.00 (0.00–0.00)	0.015	0.014	0.429	0.175
***Maxillary sinus left***	2.00 (1.00–2.00)	2.00 (2.00–2.00)	0.00 (0.00–0.00)	0.00 (0.00–1.00)	0.015	0.015	0.175	0.286
***Anterior ethmoid right***	1.00 (1.00–2.00)	2.00 (1.00–2.00)	0.00 (0.00–0.00)	1.00 (0.00–1.00)	0.015	0.023	0.061	0.228
***Anterior ethmoid left***	1.00 (1.00–2.00)	2.00 (2.00–2.00)	0.00 (0.00–0.00)	0.00 (0.00–1.00)	0.014	0.014	0.306	0.197
***Posterior ethmoid right***	0.00 (0.00–0.00)	1.00 (1.00–1.00)	0.00 (0.00–0.00)	0.00 (0.00–0.00)	0.317	0.020	0.505	0.095
***Posterior ethmoid left***	0.00 (0.00–1.00)	1.00 (1.00–1.00)	0.00 (0.00–0.00)	0.00 (0.00–0.00)	0.157	0.034	0.294	0.095
***Sphenoid right***	0.00 (0.00–1.25)	0.50 (0.00–1.25)	0.00 (0.00–0.00)	0.00 (0.00–0.00)	0.180	0.102	0.167	0.167
***Sphenoid left***	0.00 (0.00–1.25)	0.00 (0.00–2.00)	0.00 (0.00–0.00)	0.00 (0.00–0.00)	0.180	0.157	0.167	0.333
***Frontal sinus right***	2.00 (1.00–2.00)	2.00(1.75–2.00)	0.00(0.00–0.00)	0.00 (0.00–0.00)	0.038	0.034	0.167	0.167
***Frontal sinus left***	2.00 (1.00–2.00)	2.00 (1.50–2.00)	0.00 (0.00–0.00)	0.00 (0.00–0.00)	0.059	0.046	0.333	0.000
***OMC right***	0.00 (0.00–2.00)	2.00 (2.00–2.00)	0.00 (0.00–0.00)	0.00 (0.00–0.00)	0.083	0.014	0.114	0.286
***OMC left***	2.00 (0.00–2.00)	2.00 (2.00–2.00)	0.00 (0.00–0.00)	0.00 (0.00–0.00)	0.025	0.014	0.143	0.286

Abbreviations: **∞**: Median Lund-Mackay score of sinus opacification before introduction of ivacaftor scored by four otolaryngologists. #: Median Lund-Mackay score of sinus opacification after one year ivacaftor scored by four otolaryngologists. **×**: p-value of comparison between best case before and best case after one year ivacaftor. **α:** p-value of comparison between worst case before and worst case after one year ivacaftor. KCC: Kendall’s coefficient of concordance pre and post ivacaftor use. Δ: Median of best cases scored by four observers with modified Lund-Mackay score. ∑: Median of worst cases scored by four observers with the modified Lund-Mackay score. 25^th^,75^th^ IQR: 25^th^ percentile and 75^th^ percentile inter quartile range of L-M scores. OMC: ostiomeatal complex, significant: p≤0,05

### Nasal nitric oxide

**Fig 2:**
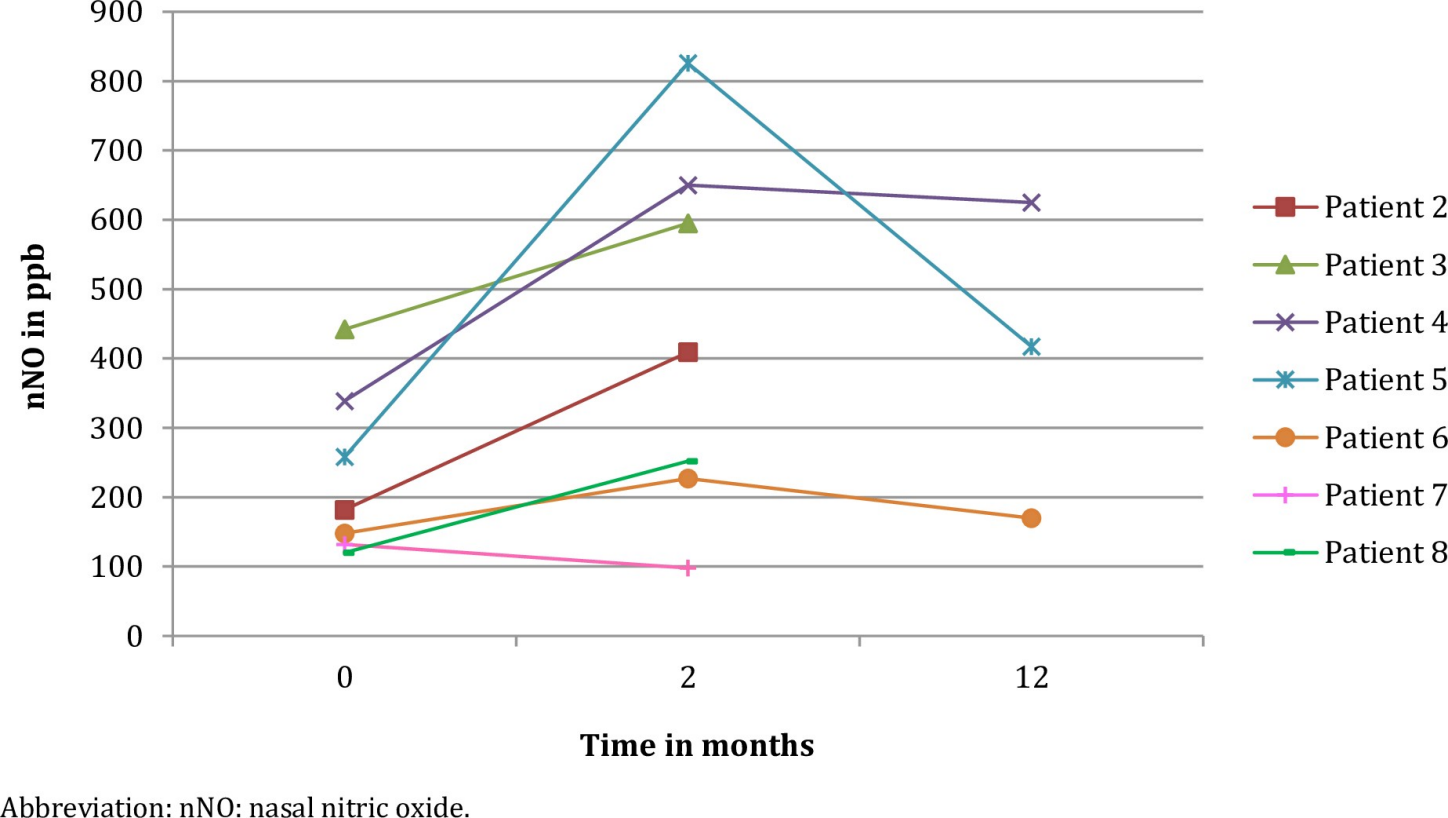
nNO measurements before and after two months and one year of ivacaftor therapy. Abbreviation: nNO: nasal nitric oxide.

Median nNO (IQR) levels increased significantly from 220,00 (136,00–341,18) to 462,84 (233,17–636,25) parts per billion (ppb) (p = 0,017) after two months of ivacaftor use, see Table 3. In four out of eight patients, nNO levels were also available after one year of ivacaftor therapy. In these four patients, from two to twelve months, nNO levels did not further increase. (583,34 (299,42–781,75) to 521,00 (231,75–689,50) ppb (p = 0,465)) (Fig 2 and Table 3).

**Table 3:** nNO measurements before and after two months and one year of ivacaftor therapy.

Compared nNO measurements	No.	Median nNO in ppb	Percentiles nNO (25^th^–75^th^)	p-value
**nNO T0**	8	220.00	136.00–341.18	0.017
**nNO T2**	8	462.84	233.17–636.25	
**nNO T0**	4	298.35	175.50–341.18	0.068
**nNO T12**	4	521.00	231.75–689.50	
**nNO T2**	4	583.34	299.42–781.75	0.465
**nNO T12**	4	521.00	231.75–689.50	

Abbreviations: nNO: nasal nitric oxide, No.: number of patients, ppb: parts per billion, significant: p ≤ 0.05, nNO T0: median nNO levels measured before introduction of ivacaftor, nNO T2: median nNO levels measured after 2 months ivacaftor, nNO T12: median nNO levels measured after 1 year ivacaftor.

### Sinonasal symptoms and nasal endoscopic findings

After treatment with ivacaftor, none of the eight included patients had symptoms of nasal obstruction, postnasal drip and facial pain. Before the use of ivacaftor four patients (50%) occasionally reported symptoms of nasal obstruction and one patient (13%) had often symptoms of nasal obstruction. Postnasal drip was reported occasionally in one patient (13%) and often in another patient (13%) before ivacaftor use. In one patient (13%) facial pain was often present before the use of ivacaftor. (Table 4). Five patients (63%) had no headaches after ivacaftor therapy compared with just two patients (25%) before ivacaftor. One patient (13%) noticed a major improvement in symptoms of anosmia or hyposmia after using ivacaftor, as this patient regained a sense of smell after three weeks of ivacaftor use. Two patients (25%) reported a marked increase of rhinorrhea symptoms after starting with ivacaftor. (Table 4 and S1 Fig) Furthermore, in three patients (38%) nasal polyps and nasal discharge resolved after the treatment with ivacaftor. Controversially, in two patients (25%) an increase of edema after using ivacaftor was reported. (Table 4 and S2 Fig)

**Table 4:** Frequency of sinonasal symptoms and endoscopic signs before and after the introduction of ivacaftor therapy.

Aspect of sinonasal disease	Pre ivacaftor	Post ivacaftor
***Rhinorrhea***		
** No symptoms, No. (%)**	4 (50)	5 (63)
** Occasionally symptoms, No. (%)**	1 (13)	0 (0)
** Often symptoms, No. (%)**	3 (38)	0 (0)
** Continuous symptoms, No. (%)**	0 (0)	2 (25)
***Nasal obstruction***		
** No symptoms, No. (%)**	3 (38)	8 (100)
** Occasionally symptoms, No. (%)**	4 (50)	0 (0)
** Often symptoms, No. (%)**	1 (13)	0 (0)
** Continuous symptoms, No. (%)**	0 (0)	0 (0)
***Postnasal drip***		
** No symptoms, No. (%)**	6 (75)	8 (100)
** Occasionally symptoms, No. (%)**	1 (13)	0 (0)
** Often symptoms, No. (%)**	1 (13)	0 (0)
** Continuous symptoms, No. (%)**	0 (0)	0 (0)
***Facial pain***		
** No symptoms, No. (%)**	6 (75)	8 (100)
** Occasionally symptoms, No. (%)**	0 (0)	0 (0)
** Often symptoms, No. (%)**	1 (13)	0 (0)
** Continuous symptoms, No. (%)**	0 (0)	0 (0)
***Headache***		
** No symptoms, No. (%)**	2 (25)	5 (63)
** Occasionally symptoms, No. (%)**	4 (50)	2 (25)
** Often symptoms, No. (%)**	2 (25)	0 (0)
** Continuous symptoms, No. (%)**	0 (0)	0 (0)
***Anosmia***		
** No symptoms, No. (%)**	4 (50)	5 (63)
** Occasionally symptoms, No. (%)**	1 (13)	0 (0)
** Often symptoms, No. (%)**	1 (13)	0 (0)
** Continuous symptoms, No. (%)**	0 (0)	0 (0)
***Nasal polyps***		
** No polyps, No. (%)**	4 (50)	7 (88)
** In middle meatus only, No. (%)**	4 (50)	1 (13)
** Beyond middle meatus, No. (%)**	0 (0)	0 (0)
***Discharge***		
** No discharge, No. (%)**	4 (50)	7 (88)
** Clear thin discharge, No. (%)**	4 (50)	0 (0)
** Thick purulent discharge, No.(%)**	0 (0)	0 (0)
***Edema***		
** Absent, No. (%)**	7 (88)	4 (50)
** Mild, No. (%)**	1 (13)	3 (38)
** Severe, No. (%)**	0 (0)	0 (0)

Abbreviations: No.: number of patients

### Discussion

This study shows that ivacaftor plays an important role in the improvement of sinonasal health in CF patients with an S1251N mutation. CT scan analysis reveals a significant decrease in opacification for the majority of paranasal sinuses one year after treatment with ivacaftor. Nasal nitric oxide levels significantly improve. Likewise, the majority of sinonasal symptoms and nasal endoscopic pathology decrease or resolve after two months of ivacaftor use.

The CT scans showed a considerable decrease in sinuses opacification for all paranasal sinuses in all patients after ivacaftor use during a period of one year. For the maxillary sinus, anterior ethmoid and specific parts of the frontal sinus and ostiomeatal complex, we notice a statistically significant decrease. However, this was not the case for the little improvement of opacification seen in the posterior ethmoid and sphenoid. These results are in accordance with the study of Sheikh et al. 2015, which shows significant improvement, measured by computed tomography (CT) scan, after treatment with ivacaftor in twelve G551D-mutated CF patients.[27]

The significant increase of nNO levels after two months of ivacaftor use can possibly be explained by the decrease of thick mucus lining, nasal polyps or inflammation and thereby decrease of obstruction of the paranasal sinuses and upper airways. That this finding is reversible after CFTR modulation with ivacaftor is a novelty an should be emphasized. Unfortunately, due to the retrospective analysis, nNO measurements of only four of the eight included patients are available after one year of ivacaftor use. In three patients the nNO level is lower after one year ivacaftor use in comparison with the nNO level after two months of ivacaftor therapy. A possible reason for the decrease in nNO level is that nasal medication was stopped because of major improvements patients experienced with regards to their sinonasal symptoms. Alternatively, it could be due to a variation in measurement in a small group of patients.

Additionally, sinonasal symptoms improved while treated with ivacaftor. All included patients react positively with regards to their sinonasal symptoms after the introduction of ivacaftor. Notably, shortly after the start of ivacaftor patients often report an increase in mucus production, sometimes accompanied by headaches. Usually these complaints disappear after a couple of days and patients report major improvements in physical energy and lack of nasal obstruction with absence of thick mucus. Some of the patients even stop their nasal medication because of these major improvements. Besides headaches, increase of sputum, stomach pain, nausea and vomiting, no major adverse effects of ivacaftor were reported. Our results are consistent with previous reports. For example, Chang et al. 2015, describes the reversal of sinonasal symptoms in a G551D/P205S-mutated patient after the use of ivacaftor.[28] Just as in 2015, a case report from Vreede et al. which describes the improvement in sinonasal pathology in an S1251N-mutated CF patient treated with the potentiator ivacaftor.[29]

The strength of this study is that the CT scans are independently scored by four otolaryngologists. Looking at the interrater variability, the reliability of this modified L-M scoring system to score CT scans by different otolaryngologists, seems to be moderate. Another strength of this study is that this is the first study that investigates the sinonasal condition before and after the use of ivacaftor by means of four different paranasal outcome measurements.

Some limitations need to be mentioned. Due to the design of our study, information about sinonasal symptoms is retrospectively extracted from the medical records. Consequently, this leads to unequally documented symptoms for different patients, and a high amount of missing values. No standardized questionnaires, like the Sino-Nasal Outcome Test -20 (SNOT-20), are used for the documentation of sinonasal symptoms of included patients in a fixed, structured way.[32,44–47] Additionally, since previous studies have shown that CF patients often underreport their CF-related chronic sinonasal symptoms, it could well be possible that not all sinonasal symptoms of the patients are documented correctly. [5,8,44,48]

As the life expectancy of CF patients continues to increase, the number of patients seeking treatment for CF-related chronic sinus disease will most likely continue to increase.[49] In the future, studies should measure sinonasal outcome parameters, like sinus CT opacification, nNO levels and sinonasal questionnaires to gain a better understanding of the role of CFTR modulators in the treatment and follow-up of sinonasal pathology in patients with CF. We therefore recommend to measure nNO concentration levels and to use standardized scoring systems for sinus CT scan analyses, because this can help to document sinonasal health in patients with CF.

Avenues for future research could be to combine two potentiators in S1251N mediates patients to see if there is an increase CFTR mediated chloride secretion and thereby a better treatment for Cystic Fibrosis patients. Or are there similar effects with other sorts of potentiator drugs?

## Conclusion

Treatment with ivacaftor significantly decreases sinus opacification as seen on sinus CT, increases nNO concentration levels and improves sinonasal symptoms and endoscopic findings, indicating a positive impact on sinonasal health in CF patients with an S1251N mutation.

## Supporting information

S1 Table Upper and lower airway cultures before and after ivacaftor therapy. (DOCX)

S2 Table CT scan scoring by the first otolaryngologist before and after one year ivacaftor. (DOCX)

S3 Table CT scan scoring by the second otolaryngologist before and after one year ivacaftor. (DOCX)

S4 Table CT scan scoring by the third otolaryngologist before and after one year ivacaftor. (DOCX)

S5 Table CT scan scoring by the fourth otolaryngologist before and after one year ivacaftor. (DOCX)

S1 Fig Frequency of sinonasal symptoms before and after ivacaftor therapy. (DOCX)

S2 Fig Frequency of endoscopic signs before and after ivacaftor therapy. (DOCX)

S3 Fig CT scans before and after one year of ivacaftor therapy. (DOCX)
