# Supplementary material for: The impact of ivacaftor on sinonasal pathology in S1251N-mediated cystic fibrosis patients
Source: PLoS One. 2020 Jul 20;15(7):e0235638. doi: 10.1371/journal.pone.0235638 (PMC7371187; doi:10.1371/journal.pone.0235638)
Supplement: S1 Table — (DOCX) [file pone.0235638.s001.docx]

**S1 Table. Upper and lower airway cultures before and after ivacaftor therapy**

|  | Upper airway culture^*^ | | Lower airway culture^*^ | |
| --- | --- | --- | --- | --- |
| Patient | **Pre ivacaftor** | **Post ivacaftor** | **Pre ivacaftor** | **Post ivacaftor** |
| 1 | No PMO, yeast, fungus | No PMO, yeast, fungus | No PMO, yeast, fungus | No PMO, yeast, fungus |
| 2 | Pseudomonas aeruginosa 10-100cfu, E. coli <10cfu | No PMO, yeast, fungus | Candida species <10cfu, pseudomonas aeruginosa 10-100cfu Aspergillus fumigatus, Achromobacter xylosoxidans> 100cfu | Aspergillus fumigatus, candida species, Pseudomonas aeruginosa 10-100cfu |
| 3 | Pseudomonas aeruginosa 10-100cfu | No PMO, yeast, fungus | Aspergillus fumigatus, pseudomonas aeruginosa >100cfu | Candida species,  Staphylococcus aureus 10-100 cfu, pseudomonas aeruginosa 10-100 |
| 4 | No PMO, yeast, fungus | Candida species | Aspergillus fumigatus, Stenotrophomonas maltophilia  10-100cfu | Candida species |
| 5 | No PMO, yeast, fungus | No PMO, yeast, fungus | No PMO, yeast, fungus | No PMO, yeast, fungus |
| 6 | Pseudomonas aeruginosa <10 cfu, | Staphylococcus aureus 10-100cfu, Alternaria species | Candida albicans 10-100,  Aspergillus fumigatus | Staphylococcus aureus 10-100 cfu, Streptococcus agalactiae (groep B) >100 cfu Haemophilus influenzae 10-100 cfu, Candida albicans <10 Pseudomonas aeruginosa <10 |
| 7 | No PMO, yeast, fungus | No PMO, yeast, fungus | Candida species <10 | Candida albicans 10-100 cfu, Staphylococcus aureus <10 cfu, |
| 8 | Staphylococcus aureus<10cfu | No PMO, yeast, fungus | Staphylococcus Aureus>100, Aspergillus fumigatus | Candida species,  Staphylococcus aureus <10 cfu |

Abbreviations: *: Upper airway culture are nasopharynx cultures, lower airway culture are oropharynx and sputum cultures. PMO: pathological micro-organisms, cfu: colony forming unit
