# Supplementary material for: The impact of ivacaftor on sinonasal pathology in S1251N-mediated cystic fibrosis patients
Source: PLoS One. 2020 Jul 20;15(7):e0235638. doi: 10.1371/journal.pone.0235638 (PMC7371187; doi:10.1371/journal.pone.0235638)
Supplement: S4 Table — (DOCX) [file pone.0235638.s004.docx]

**S4 Table. CT scan scoring by the third otolaryngologist before and after one year ivacaftor**

| Patient | | Maxilla | | Anterior ethmoid | | Posterior ethmoid | | Sphenoid | | Frontal | | OMC | | Total  (Total L-M score/total present sinuses) | Absence of sinuses | |
| --- | --- | --- | --- | --- | --- | --- | --- | --- | --- | --- | --- | --- | --- | --- | --- | --- |
|  |  | *R* | *L* | *R* | *L* | *R* | *L* | *R* | *L* | *R* | *L* | *R* | *L* |  | *R* | *L* |
| 1 | pre | 1 | 1 | 2 | 2 | 1 | 1 | 1 | 0 | 2 | 2 | 2 | 2 | 17/24 | - | - |
|  | post | 0 | 0 | 1 | 0 | 0 | 0 | 0 | 0 | 2 | 0 | 0 | 0 | 3/24 | - | - |
| 2 | pre | 2 | 2 | 2 | 1 | 0 | 0 | 0 | 0 | - | - | 2 | 2 | 11/20 | Fr. | Fr. |
|  | post | 0 | 0 | 0 | 0 | 0 | 0 | 0 | 0 | - | - | 0 | 0 | 0/20 | Fr. | Fr. |
| 3 | pre | 2 | 2 | 1 | 1 | 1 | 1 | 1 | 2 | 2 | - | 0 | 2 | 15/22 | - | Fr. |
|  | post | 0 | 0 | 0 | 0 | 0 | 0 | 0 | 0 | 0 | - | 0 | 0 | 0/22 | - | Fr. |
| 4 | pre | 2 | 2 | 2 | 1 | 0 | 0 | 0 | 0 | 2 | 2 | 2 | 2 | 12/24 | - | - |
|  | post | 0 | 0 | 0 | 0 | 0 | 0 | 0 | 0 | 0 | 0 | 0 | 0 | 0/24 | - | - |
| 5 | pre | 1 | 2 | 1 | 1 | 0 | 0 | - | - | 1 | - | 2 | 2 | 10/18 | Sph. | Sph, Fr. |
|  | post | 0 | 1 | 0 | 0 | 0 | 0 | - | - | 0 | 0 | 0 | 0 | 1/20 | Sph. | Sph. |
| 6 | pre | 2 | 2 | 2 | 2 | 0 | 0 | 2 | 1 | 2 | - | 0 | 0 | 11/22 | - | Fr. |
|  | post | 1 | 1 | 2 | 2 | 1 | 1 | 1 | 0 | 2 | 2 | 2 | 2 | 17/24 | - | - |
| 7 | pre | 0 | 0 | 1 | 0 | 0 | 0 | 0 | 0 | 2 | 0 | 0 | 0 | 3/24 | - | - |
|  | post | 2 | 2 | 2 | 1 | 0 | 0 | 0 | 0 | - | - | 2 | 2 | 11/20 | Fr. | Fr. |

Abbreviations: pre: before Ivacaftor, post: after start of ivacaftor therapy, R: right side sinus, L: left side sinus. OMC = ostiomeatal complex, Fr.: frontal sinus, Sph.: sphenoid. Sinuses grading system, Lund-Mackey score: 0 = normal, 1 = partial opacification 2= total opacification. OSM: 0 = patent, 2 = occluded.
