# Supplementary material for: The impact of ivacaftor on sinonasal pathology in S1251N-mediated cystic fibrosis patients
Source: PLoS One. 2020 Jul 20;15(7):e0235638. doi: 10.1371/journal.pone.0235638 (PMC7371187; doi:10.1371/journal.pone.0235638)
Supplement: S2 Fig — (DOCX) [file pone.0235638.s007.docx]

**S2 Fig. Frequency of endoscopic signs before and after ivacaftor therapy**
