# Supplementary material for: The impact of ivacaftor on sinonasal pathology in S1251N-mediated cystic fibrosis patients
Source: PLoS One. 2020 Jul 20;15(7):e0235638. doi: 10.1371/journal.pone.0235638 (PMC7371187; doi:10.1371/journal.pone.0235638)

**S3 Fig. – CT scans before and after one year of ivacaftor therapy**

**Before ivacaftor therapy After one year of ivacaftor therapy**

**
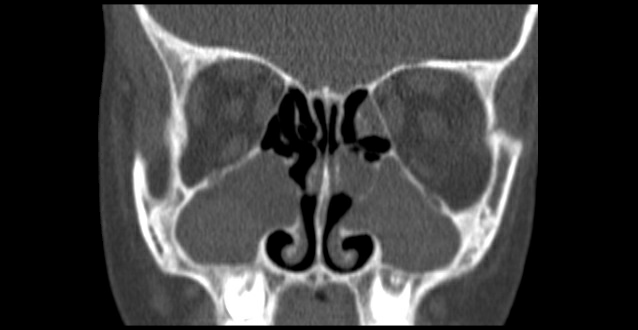

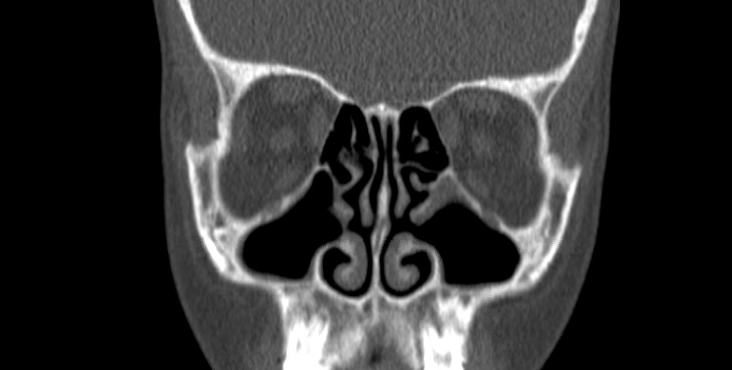
1a. 1b.**

**
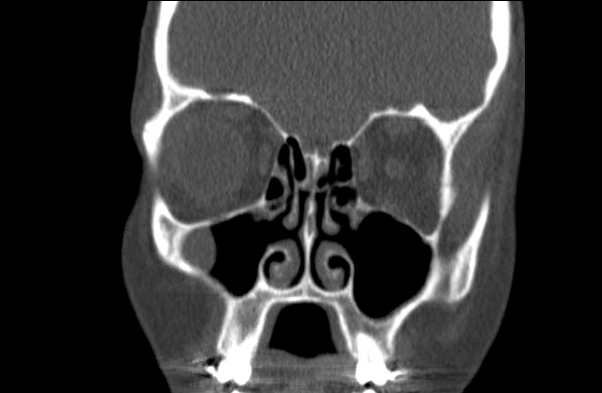
2a. 2b.**

**
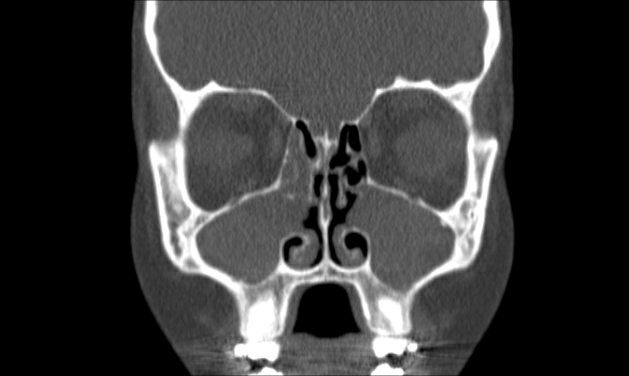
**

**
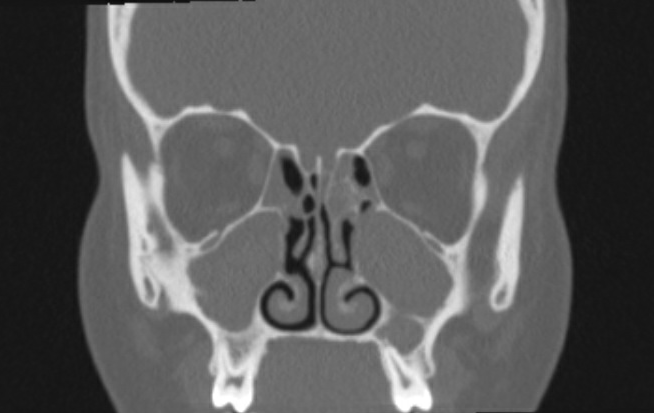

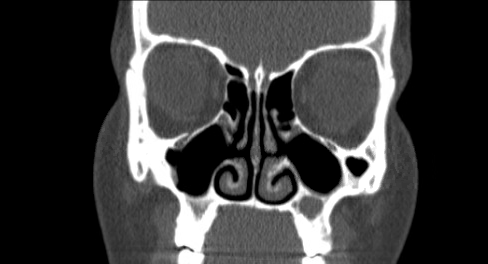
3a. 3b.**

**4a. 4b.**


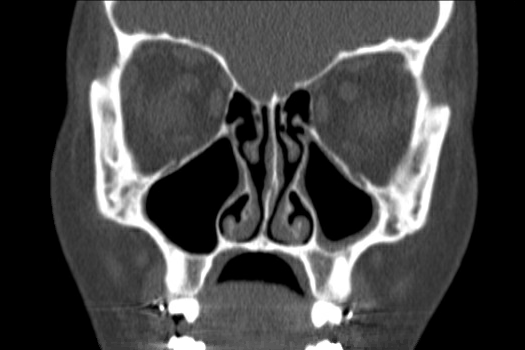

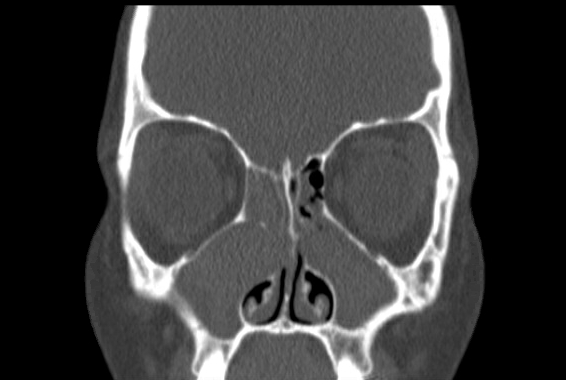


**5a. 5b.**


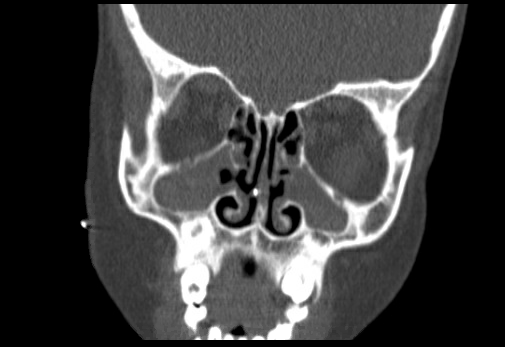

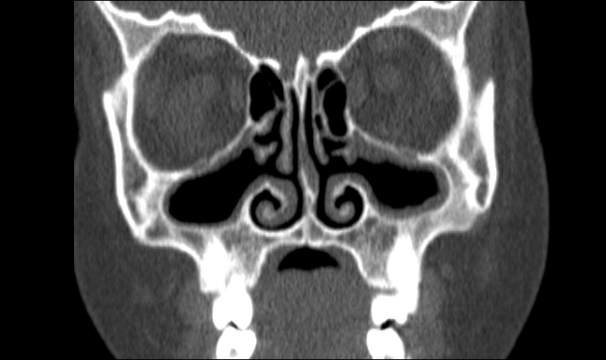


**6a. 6b.**


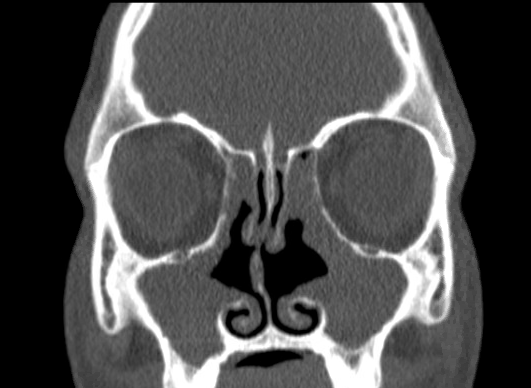

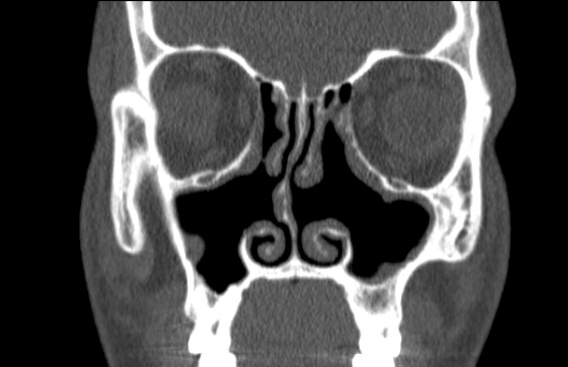


**7a. 7b.**


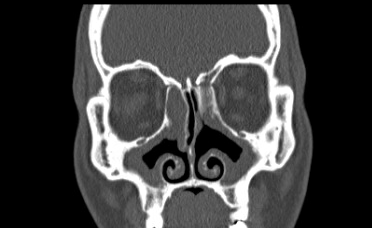

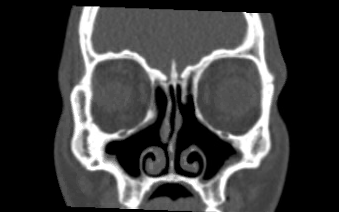

Supplement: S3 Fig — (DOCX) [file pone.0235638.s008.docx]
